# Differential Role of Leptin and Adiponectin in Cardiovascular System

**DOI:** 10.1155/2015/534320

**Published:** 2015-05-03

**Authors:** C. M. Ghantous, Z. Azrak, S. Hanache, W. Abou-Kheir, A. Zeidan

**Affiliations:** ^1^Department of Anatomy, Cell biology and Physiology, American University of Beirut, DTS-255, P.O. Box 11-0236, Beirut 1107-2020, Lebanon; ^2^Department of Pharmacology and Toxicology, American University of Beirut, DTS-255, P.O. Box 11-0236, Beirut 1107-2020, Lebanon

## Abstract

Leptin and adiponectin are differentially expressed adipokines in obesity and cardiovascular diseases. Leptin levels are directly associated with adipose tissue mass, while adiponectin levels are downregulated in obesity. Although significantly produced by adipocytes, leptin is also produced by vascular smooth muscle cells and cardiomyocytes. Plasma leptin concentrations are elevated in cases of cardiovascular diseases, such as hypertension, congestive heart failure, and myocardial infarction. As for the event of left ventricular hypertrophy, researchers have been stirring controversy about the role of leptin in this form of cardiac remodeling. In this review, we discuss how leptin has been shown to play an antihypertrophic role in the development of left ventricular hypertrophy through *in vitro* experiments, population-based cross-sectional studies, and longitudinal cohort studies. Conversely, we also examine how leptin may actually promote left ventricular hypertrophy using *in vitro* analysis and human-based univariate and multiple linear stepwise regression analysis. On the other hand, as opposed to leptin's generally detrimental effects on the cardiovascular system, adiponectin is a cardioprotective hormone that reduces left ventricular and vascular hypertrophy, oxidative stress, and inflammation. In this review, we also highlight adiponectin signaling and its protective actions on the cardiovascular system.

## 1. Introduction

According to the Centers for Disease Control and Prevention (CDC), more than one-third of U.S. adults are obese. Generally, obesity is associated with high levels of the circulating hormone leptin (hyperleptinemia) and low levels of adiponectin [1–3]. Leptin and adiponectin are cytokines produced excessively by adipocytes, hence the name “adipokines.” Leptin is thought to be responsible for several cardiovascular diseases associated with obesity, while adiponectin is considered to be cardioprotective. This review covers the relationship between leptin, adiponectin, and the cardiovascular system.

## 2. Leptin

Leptin is a 16 kDa protein which functions as a satiety factor. It is secreted by adipocytes and binds to the hypothalamic leptin receptor (Ob-R) to enhance metabolism and reduce appetite [4], thereby increasing energy expenditure and decreasing energy intake. It is a product of the* ob* gene [5] and is associated with obesity, since a higher adipose tissue mass results in elevated leptin levels [6].

Leptin is also produced by other cells besides adipocytes, such as cardiomyocytes and vascular smooth muscle cells (VSMC) [7, 8]. Several studies have shown that the functional leptin receptor is also found in a variety of organs such as the heart, liver, kidneys, and pancreas [9–13]. It is located on cardiomyocytes [14], vascular smooth muscle cells [5], endothelial cells [15], myometrium [16], and cerebral and coronary vessels [17, 18]. Therefore, this hormone has a wide range of pleiotropic effects, affecting the cardiovascular, nervous, immune, and reproductive systems [19–21].

Leptin circulates in the blood at a level of 5 to 15 ng/mL in lean individuals [22]. This level may reach up to 50 ng/mL in obese individuals, due to their higher adipose tissue mass. Glucocorticoids and insulin act on adipocytes to increase leptin expression, possibly explaining the reason for increased leptin levels observed in obesity [23].On the other hand, fasting, testosterone, and thyroid hormone lead to a reduction in leptin expression [23, 24].

### 2.1. Leptin Signaling

#### 2.1.1. Leptin Receptor

Although well-known as a product of adipocytes, leptin is also produced by a variety of different tissues and has many functions other than being a satiety factor [8, 14, 25]. In the murine model, the leptin receptor Ob-R has six isoforms, Ob-Ra to Ob-Rf, which are strongly related to class I cytokine receptor family. They are alternatively spliced but contain the same ligand-binding domain [26]. Ob-Re is a soluble receptor secreted in the blood that binds to circulating leptin in order to maintain the concentration of free leptin [17, 26, 27]. The other Ob-R receptors are transmembrane proteins on the plasma membrane. Ob-Ra, c, d, f are short isoforms. Ob-Rb is the long, functional isoform, responsible for the intracellular signaling effects of leptin [26]. Binding of leptin to the Ob-Rb receptor activates the Janus-activated kinase (JAK) signal transduction pathway, Signal Transducers and Activators of Transcription (STAT) pathway, insulin receptor substrate, and Mitogen-Activated Protein Kinase (MAPK) pathway [28].

#### 2.1.2. Leptin Signaling Pathways

*Leptin and the JAK/STAT Pathway*. The JAK/STAT pathway is the best illustrated pathway in leptin signaling [29]. When leptin binds to Ob-Rb, this receptor undergoes homooligomerization [30, 31] and then binds to JAK2 [32]. This leads to autophosphorylation of JAK2 and the phosphorylation of Tyr985, Tyr1077, and Tyr1138 on Ob-Rb [30, 32–35]. Phosphorylation of Tyr1138 residue on Ob-Rb recruits STAT3 proteins to the Ob-Rb/JAK2 complex and leads to tyrosine phosphorylation of STAT3 proteins, which form dimers and translocate to the nucleus in order to activate transcription of target genes. One of these genes is a member of the suppressors of the cytokine signaling family (SOCS3) [33, 36, 37].

SOCS3 binds to Tyr985 and other sites within the Ob-Rb/JAK2 complex which inhibits leptin signaling [38, 39]. JAK2 phosphorylates Tyr985 and leads to the phosphorylation of the SH2 (*src* homology 2) domain of the tyrosine phosphatase SHP-2 (*src* homology 2-containing tyrosine phosphatase), which in turn activates the extracellular signal-regulated kinase (ERK) signal transduction pathway [33]. Moreover, SHP-2 overexpression blunts SOCS3-mediated inhibition, possibly through competitive binding to Tyr985 [38].

JAK2 autophosphorylation may also lead to phosphorylation of insulin receptor substrate proteins, which activate the PI3K signaling pathway [40, 41]. In the heart, both the leptin-activated ERK and PI3K pathways are crucial for proliferation of cardiomyocytes and for the protection of cardiac tissue from ischemia/reperfusion injury [42, 43]. 

*Leptin and the MAPK Pathway*. Another major pathway activated by leptin binding to its Ob-Rb receptor is the MAPK pathway. After agonist binding, homooligomerization of the receptor, JAK2 recruitment, and autophosphorylation, the Tyr985 on Ob-Rb is phosphorylated. This recruits SHP-2 and Grb-2 (growth factor receptor-bound protein 2) to phosphorylate and activate ERK1/2 of the MAPK family [33]. ERK1/2 activation ultimately leads to the expression of specific target genes, such as* c-fos* and* egr-1*, which promote proliferation and differentiation [28, 38, 44].

Activation of ERK1/2 may also occur independently of Tyr985. In this case, JAK2 binds to the SH2 domain of SHP-2 [33, 45]. Thus, both the short leptin receptor isoform Ob-Ra and the long receptor isoform Ob-Rb can activate MAPK, but to a lesser extent by Ob-Ra [17, 33]. Another protein that contains SH2 domain and associates with Grb-2 is SHC, which has been shown to phosphorylate tyrosine after leptin agonist binding [46]. Leptin-induced phosphorylation of STAT3 and ERK1/2 has been studied in isolated adult C57BL/6 mouse cardiomyocytes, with maximal activation observed at 15 minutes after leptin treatment. Leptin-deficient* ob/ob* mice treated with leptin for four weeks also exhibited elevated STAT3 and ERK1/2 phosphorylation in their cardiac tissue, but the same treatment in Ob-Rb-deficient* db/db* mice did not lead to STAT3 and ERK1/2 phosphorylation [47].

Leptin has been shown to also lead to phosphorylation of p38 MAPK. The *α* and *β* isoforms of p38 MAPK are widely distributed and found at relatively high levels in the heart [48]. Leptin-induced p38 MAPK activation is associated with the onset of hypertrophy and programmed cell death in cardiomyocytes and rat vascular smooth muscle cells [14, 42, 49].

*Leptin and the Rho Pathway*. Under the hypertension-induced force of mechanical stretch, guanine nucleotide exchange factors exchange GDP for GTP on the guanine nucleotide (GTP) binding protein RhoA, thereby activating it. RhoA then activates Rho kinases (ROCK), which activates LIM kinase (LIMK) [50]. LIMK phosphorylates cofilin, inactivating this actin depolymerizing protein and leading to the accumulation of F-actin and depletion in G-actin [51]. When present at normal physiological levels, G-actin attenuates hypertrophy by inhibiting transcription factors like serum response factor (SRF) which upregulate hypertrophic gene expression [52, 53]. Thus, the activation of the RhoA/ROCK pathway leads to a reduction in G-actin levels, promoting vascular remodeling and hypertrophy.

### 2.2. Leptin and Cardiovascular Disease

**Figure 1:**
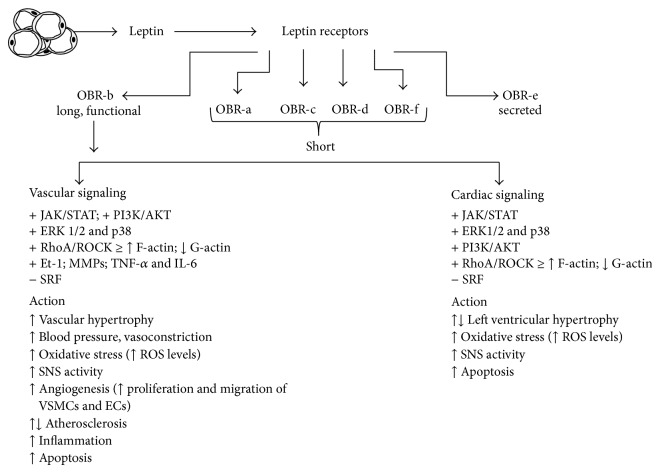
Summary of molecular signaling responses to leptin and their cardiac and vascular effect. ↑ or + represents activation of protein or effect whereas ↓ or − indicates inhibition. See text for more information.

Several studies have revealed numerous effects of leptin on the cardiovascular system [20, 54, 55]. In this review, we will discuss the effect of leptin on the cardiac and vascular system (Figure 1), focusing on cardiac hypertrophy, angiogenesis, the vasoactive response, blood pressure, and atherosclerosis.

#### 2.2.1. Cardiac Hypertrophy

The heart increases its mass as a compensatory mechanism for a hemodynamic overload. Since cardiomyocytes become terminally differentiated early in life, the increase in cardiac mass is due to hypertrophy of the myocytes rather than hyperplasia. In situations of pressure overload, such as conditions of hypertension or aortic stenosis, myocyte width increases due to the parallel addition of sarcomeres, which in turn increases wall thickness. This kind of remodeling is concentric hypertrophy (increase in wall thickness/chamber dimension ratio) [56].

Left ventricular hypertrophy (LVH) occurs when the myocardium of the left ventricle of the heart enlarges. The left ventricle is the chamber which pumps oxygenated blood into the aorta, which in turn carries and delivers blood to the various tissues and organs. Left ventricular hypertrophy generally develops in response to factors like age, hypertension, aortic valve stenosis, and obesity [57]. As the workload increases, the walls of the ventricle grow thicker and lose elasticity. This remodeling may lead to an increased risk of cardiovascular diseases [58], such as heart failure, arrhythmia, ischemic heart disease, or myocardial infarction [56, 59].

The functional leptin receptor Ob-Rb is found in the myocardium [60], allowing leptin to exert its actions on the heart. Studies have shown a direct link between leptin and myocardial structure remodeling. The role of leptin in the development LVH has been examined, with some researchers believing that leptin promotes LVH, while others strongly believing that leptin attenuates it [58]. 

*Leptin as Antihypertrophic Factor*. An interesting study by Barouch et al. examined the role of leptin as an antihypertrophic hormone in the heart. They studied the development of LVH in the leptin-lacking* ob/ob* and functional leptin receptor-lacking* db/db* mice. These morbidly obese mice exhibited a significant increase in left ventricular (LV) mass and LV wall thickness by 6 months of age [61] as seen by echocardiographic examination, indicating that they had developed LVH. Since blood pressure can lead to LVH, they measured the systolic blood pressure, LV end-diastolic pressure, and heart rate in order to adjust for these factors in case they were dissimilar between these mice and their littermate controls; they observed that they were not different [61]. Hence, hypertension was not the cause of the increase in LV wall thickness and LV mass.

Histological examinations were also made on the cardiomyocytes from* ob/ob *and* db/db *mice to visually evaluate hypertrophy. Clear myocyte hypertrophy was seen in the hearts of the* ob/ob *and* db/db *mice compared to the myocytes of wild type mice, with larger cellular diameter and distorted nuclear architecture [61]. However, no significant interstitial fibrosis, metabolic inclusions of the cytoplasm, or myocardial adipose infiltration were observed [61].

Since a lack of leptin or leptin signaling seemed to result in LVH, Barouch et al. went on to replete these* ob/ob* mice with leptin to study whether leptin had antihypertrophic ability. They had 3 groups of mice: leptin-infused mice, pair-fed mice (on a diet to lose weight), and control mice. After almost 6 weeks, the pair-fed mice had lost a significant amount of weight, as did the leptin-infused mice [61], attributable to leptin's neurohormonal ability to decrease appetite and increase energy expenditure. Interestingly, there was a full reversal of LVH in the leptin-infused mice as seen by both echocardiographic evaluation and histological analysis [61]. Their LV wall thickness returned to normal, and the LV mass significantly decreased [61]. Although the pair-fed mice lost as much weight as the leptin-infused mice, they did not have a reduction in LV wall thickness and LV mass, a similar observation to the controls [61]. Hence, weight loss alone (without leptin administration) did not reverse LVH. These results indicated not only that the LVH seen in* ob/ob* mice was simply due to their obesity, but also that leptin depletion was a significant cause.

The study by Barouch et al. concluded that leptin has a direct antihypertrophic role on the heart, independent of weight loss [61]. However, their studies were done on mice. The next step should be to measure LV mass, thickness, and other indicators of LVH in humans, preferably those born with a mutant gene for leptin, in which they cannot produce this hormone. Few families have been found to have this genetic abnormality in the leptin gene. They were unable to produce the leptin hormone, and thus they were hyperphagic and obese. After examination, they were treated with leptin, thereby losing weight and significantly improving in overall health [62, 63]. Perhaps their LV mass and thickness before leptin administration should have been measured and compared to these corresponding parameters after leptin treatment. If LVH is reduced after leptin repletion, we can conclude with further confidence that leptin directly reverses LVH in the heart.

Another confusing insight in the suggestion that leptin contributes to the reversal of LVH is that hypertension is known to lead to LVH, but hypertension is associated with increased levels of leptin [64]. This implies that leptin levels are perhaps directly associated with LVH. Also, treating leptin deficient* ob/ob* mice with leptin should elevate their blood pressure, which in turn would be a mechanical cause for the development of LVH. Barouch et al. examined LV mass, thickness, and cardiomyocyte hypertrophy after 6 weeks of leptin repletion and observed that these factors were diminished as a result of leptin treatment. However, continuous leptin administration for a period longer than only 6 weeks could possibly lead to actions of leptin besides those of being an antihypertrophic factor, but rather perhaps a hypertrophic factor.

A population-based cross-sectional study in support of the antihypertrophic effect of leptin was done by Pladevall et al. in rural Spain. They studied 410 overweight adults and focused on the correlations between plasma leptin levels and LV mass index (LVMI) and sum of wall thicknesses (SWT), both parameters of LVH. They adjusted for several factors like systolic blood pressure, body mass index, gender, insulin resistance, and age and used a multivariate linear regression model that showed that fasting leptin was inversely and significantly related to LVMI [65]. Leptin levels were also inversely associated with SWT, but not significantly. Among the participants, there was a subgroup of hypertensive patients. Within this subgroup, there was also a negative association of fasting leptin with both LVMI and SWT [65]. Similar to Barouch et al., this study suggests an inverse association between leptin levels and LVH, using the parameters of LVMI and SWT.

This study showed a negative correlation between leptin levels and SWT and LVMI in all body mass index strata, with the effect being most prominent in nonobese individuals [65]. This implies that the higher leptin levels seen in obese individuals should result in lower SWT and LVMI, but obesity is associated with LVH [57]. The only explanation is leptin resistance, but this would mean a positive association between leptin levels and SWT and LVMI in the group of obese individuals. However, a negative correlation was seen. The researchers of this study were unable to establish a temporal relationship between leptin levels and LVH due to the cross-sectional nature of this study [65], and so prospective cohort studies are required to portray the temporal sequence of this inverse relationship. Moreover, leptin levels, LVMI, and SWT were not measured at the same time [65].

The latest study examining the protective role of leptin against LVH was based on a Multi-Ethnic Study of Atherosclerosis (MESA). This longitudinal cohort study of multiethnic groups used data collected from 1,464 participants who had baseline MRI scans of the heart and leptin concentration data [66]. After adjusting for age, weight, height, race, and gender, Allison et al. performed multivariate linear regression modeling which revealed that a 1-SD increment in leptin was significantly inversely associated with LV mass, LV volume, and odds ratio for LVH incidence [66]. Thus, this recent cross-sectional study of a multiethnic group revealed that leptin was significantly associated with reduced LV mass, LV volume, and odds for the occurrence of LVH.

This interesting study is strengthened by the large size of the sample and multiethnic nature of the group. Allison et al. attributed the antihypertrophic effects of leptin to leptin-induced minimization of triglyceride deposition in the myocardium [66]. They believe that leptin resistance in obesity would prevent the high levels of leptin from inhibiting deposition of triglycerides in the heart. However, this study was a cross-sectional study design and only few subjects belonging to the highest levels of obesity were recruited [66]. Moreover, the researchers used a novel method for measuring LV mass and volume by MRI, which resulted in more artifact and signal-to-noise issues than other protocols [63]. This could possibly influence measurements of LV volume [66]. 

*Leptin as a Prohypertrophic Factor*. Since obesity is associated with LVH [57] and leptin levels are significantly increased in obesity, many researchers believe that leptin actually contributes to LVH.* In vitro* studies done on neonatal rat ventricular myocytes exposed to 3.1 nmol/L of leptin for 24 hours significantly increased cell surface area by 42% [14]. This concentration of leptin corresponds to the average leptin concentration seen in obese individuals [67]. The leptin-induced hypertrophic response was mediated by phosphorylation and subsequent activation of the MAP kinases p38 and ERK1/2, with acute responses of peak stimulation after 5–10 minutes of leptin administration [14].

In order to rule out the possibility of osmosis-induced hypertrophy, the researchers tested for protein synthesis by leucine incorporation. Leptin treatment was found to increase protein synthesis by 32% [14]. Leptin also significantly increased expression of *α*-skeletal actin and myosin light chain-2 (MLC-2), both upregulated in cardiac hypertrophy. This study underscores leptin's ability to induce ventricular hypertrophy at concentrations well within those of obese individuals, proposing a potential direct link between obesity-associated hyperleptinemia and increased risk of cardiovascular diseases, particularly those associated with hypertrophy.

Leptin is thought to induce cardiomyocyte hypertrophy through its signaling via the PI3K-AKT and MAP kinases such as ERK1/2 and p38 [14, 43, 68, 69]. Transgenic mice with dominant-negative mutants of PI3K in their myocardia had smaller hearts than controls [68], while those expressing constitutively active forms of PI3K or AKT had larger hearts than controls [68, 69]. Also, rapamycin, which interferes with PI3K signaling, has been shown to attenuate cardiac hypertrophy [70]. Inhibitors of ERK 1/2 and p38 activation have also been shown to reduce leptin-induced cardiomyocyte hypertrophy [14, 43].

A human study was done by Perego et al. to examine leptin's role in developing LVH by calculating LV mass in obese individuals undergoing bariatric surgery of laparoscopic adjustable gastric banding (LAGB) [71]. LAGB is a safe, minimally invasive surgery that reduces the size of the stomach in order to induce weight loss. The patients they chose for this study were morbidly obese and normotensive, to rule out the possibility of hypertension-induced LVH. The controls were healthy, lean, normotensive people.

As expected, both leptin levels and LV mass were significantly higher in the obese individuals as compared to the lean controls. Univariate regression analysis showed a significant association between LV mass and BMI, leptin concentration, insulin, and HOMA index [71]. In addition, LV mass correlated with blood glucose and blood pressure as evaluated by electrocardiogram and electrocardiography, respectively. However, when each of these factors' contribution to LVH was individually studied, only leptin was found to be a significant determinant of LV mass increase, independent of age, gender, BMI, HOMA index, insulin, and glucose [71].

Obese patients who underwent the LAGB were reevaluated a year later, after their BMI had decreased by an average of around 20%. The LV mass of these patients decreased by about 12%, leptin by almost 47%, insulin by around 49%, and HOMA index by 56% [71]. However, the reduction in LV mass only correlated with the decrease in leptin levels at simple regression analysis [71]. At multiple regression analysis, LV mass only correlated with leptin levels. This underscores leptin's role in the development of LVH.

The study by Perego et al. was done using normotensive obese patients in order to rule out the possibility of hypertension-mediated LVH and to focus on an excess amount of leptin in developing LVH. Another study by Paolisso et al. was directed at examining the role of leptin in LVH development in insulin-resistant hypertensive patients who were not necessarily obese [72]. Chronic leptin administration increases blood pressure and heart rate through sympathetic nervous system stimulation [73, 74], which is involved in the pathogenesis of LVH [75]. They found that fasting plasma leptin levels were significantly higher in hypertensive subjects than in normotensive controls, with no change in these results after adjustment for BMI [72]. The LV mass was greater in hypertensive patients according to echocardiographic evaluation. Using multiple linear stepwise regression analysis, plasma leptin concentration was significantly and independently associated with the increase LV wall thickness in hypertensive patients [72].

LV wall thickness has been positively correlated with insulin resistance in hypertension [76], and leptin has been associated with insulin resistance [77]. Although the correlation between plasma leptin levels and LV wall thickness might be driven by insulin resistance, leptin remained independently associated with LV wall thickness in the multivariate model even after adjusting for insulin action [72]. This suggests that leptin's role in promoting LVH is at least partially independent of insulin action. Paolisso et al. attributed leptin's hypertrophic actions to its ability to activate the sympathetic nervous system, which in turn can lead to LVH in hypertensive patients [75, 78].

#### 2.2.2. Vascular Action of Leptin

Leptin exerts several actions on the vascular system. It contributes to vascular remodeling, hypertrophy, and angiogenesis. It also plays an important role in hypertension. The involvement of leptin in promoting atherosclerosis is still controversial, with many researchers supporting leptin's role as an atherogenic factor, while others studying its antiatherogenic properties. 

*Vascular Wall*. Zeidan et al. have shown that leptin is secreted as a result of the hypertension-mimicking mechanical stretch of the rat portal vein [8], suggesting that leptin expression is increased in hypertension. They also observed that leptin directly leads to hypertrophy of the rat portal vein wall [8]. With respect to the molecular mechanisms underlying the leptin-induced vascular remodeling, different pathways and signaling cascades are involved, such as the RhoA/ROCK pathway, PI3K/AKT pathway, and the MAP Kinases [8, 51]. 

*Blood Pressure*. Not only are leptin concentrations directly increased as a result of hypertension [8], leptin itself contributes to the development of hypertension. Rodents exposed to intravenous infusion and intracerebroventricular leptin administration demonstrated increased arterial pressure and heart rate [74, 79], while blocking the adrenergic system diminished leptin-induced hypertension [80]. Other mechanisms, besides increased sympathetic activity associated with hyperleptinemia, may also be responsible for the development of obesity-related hypertension. For instance, leptin leads to the secretion of proinflammatory cytokines, such as TNF-*α* and IL-6, and to the generation of reactive oxygen species (ROS) in endothelial cells [81, 82], both promoters of hypertension [83]. Leptin has also been shown to augment the release of the vasoconstrictor endothelin-1 (ET-1) primarily in endothelial cells, but also in cardiomyocytes, fibroblasts, and macrophages [84], also leading to an elevation in blood pressure.

During hypertension, ROS production is increased. This in turn leads to oxidative stress and the growth, migration, and hypertrophy of VSMC [85]. Leptin is secreted in high concentrations as a result of the mechanical stretch associated with hypertension [8], and leptin alone is able to increase ROS generation [81, 86]. Hence, under conditions of hypertension and obesity, leptin concentrations are augmented, leading to an increase in ROS production and oxidative stress, which in turn lead to dysfunction and vascular remodeling through oxidative damage [87]. 

*Angiogenesis*. Another vascular effect of leptin is its ability to promote angiogenesis. For angiogenesis to occur, endothelial and vascular smooth muscle cells must migrate and proliferate. Leptin promotes this process in endothelial cells by upregulating expression of vascular endothelial growth factor (VEGF) [88] and inducing actin cytoskeleton reorganization [89]. Leptin also contributes to vascular smooth muscle cell proliferation and migration [90] through upregulating the serine/threonine kinase Akt [91], activating ERK1/2 [92, 93], and promoting reorganization of the actin cytoskeleton through the RhoA/ROCK pathway [51]. Leptin also increases the expression of matrix metalloproteinases (MMPs), thereby inducing vascular basement membrane degradation and modification of the extracellular matrix, both key events in angiogenesis [94, 95]. MMPs further lead to angiogenesis by releasing growth factors which also cause cell proliferation [96]. Moreover, leptin-induced ROS generation also contributes to angiogenesis through the ability of ROS to promote lipoprotein lipase expression from macrophages [86] and VEGF secretion by endothelial cells [97] and VSMC [98]. 

*Atherosclerosis*. Over the years, leptin has been implicated in the development of atherosclerosis due to the presence of leptin receptor in the different compartments of atherosclerotic lesions. These include endothelial cells [99], vascular smooth muscle cells [90], macrophages, and foam cells [94]. Atherosclerosis occurs with neointimal formation, which is the thickened layer of cells that have proliferated and migrated. Leptin is believed to cause atherosclerosis by promoting the proliferation and migration of vascular smooth muscle and endothelial cells, thus inducing neointimal growth [90, 100, 101].

Leptin induces proliferation of vascular smooth muscle cells by a mechanism involving stimulation of phosphatidylinositol 3-kinase (PI3K) activity [90], activation of mitogen-activated protein (MAP) kinases, and progression to S and G2/M phases [102]. It promotes the migration of vascular smooth muscle cells by activating the Rho/ROCK pathway which promotes reorganization of the actin cytoskeleton [103]. Leptin further leads to neointimal growth by stimulating platelet aggregation [104], activating monocytes [105], and regulating the immune response [106, 107]. Moreover, leptin promotes oxidative stress [81, 86], which in turn leads to vascular smooth muscle and endothelial damage [108]. This leads to the deposition of lipids within blood vessel and adhesion of macrophages and lymphocytes [108–110].

The atherogenic effect of leptin was shown by Schäfer et al. [100] using leptin deficient* ob/ob* mice. Following atherogenic high fat diet, wild type mice exhibited significant neointimal thickening after carotid artery injury [100]. On the other hand,* ob/ob* mice, although obese and hyperlipidemic, did not have a significant increase in neointimal thickness even after feeding on a high fat diet and being exposed to vascular injury [100]. The wild type and* ob/ob* mice were then treated with leptin daily for 3 weeks, which lead to a dramatic increase in lesion size and the severity of luminal stenosis after arterial injury, regardless of whether the diet was normal chow or a high fat diet [100]. Also, the vascular lesions formed in response to injury showed strong expression for the leptin receptor mRNA in the endothelial cells, vascular smooth muscle cells, and macrophages [100], indicating that leptin indeed was mediating its effect on these different components of the neointima.

Paradoxically, some researchers believe that leptin reduces atherosclerosis. In a study that involved Ins2^+/Akita^:apoE^−/−^ mice which developed type 1 diabetes, hypercholesteremia, and atherosclerosis spontaneously, severe leptin deficiency was seen compared to nondiabetic Ins2^+/+^:apoE^−/−^ mice [111]. At 13 weeks of age, the Ins2^+/Akita^:apoE^−/−^ mice were treated with leptin for 3 months. Leptin therapy significantly decreased plasma cholesterol concentrations by around 41%, mainly in LDL fractions [111]. It also substantially reduced aortic atherosclerotic lesion area in the Ins2^+/Akita^:apoE^−/−^ mice by almost 62% [111]. This study proposed that leptin treatment could improve dyslipidemia and thus attenuate atherosclerosis in cases of type 1 diabetes. However, it does not directly prove that leptin could attenuate atherosclerosis, in nondiabetics per se.

## 3. Adiponectin

Adiponectin, also termed adipocyte complement-related protein of 30 kDa (Acrp30), AdipoQ, apM1, or GBP28, is an adipokine produced and secreted exclusively by both white adipose tissue (WAT) and brown adipose tissue. It accounts for around 0.01% of the total plasma protein in humans [112]. In healthy lean individuals, the adiponectin serum levels range between 5 and 30 *μ*g/mL [112]. Adiponectin level negatively correlates with cardiovascular and metabolic disorders [112–116], indicating adiponectin's important role in the cardiovascular system. In contrast to other adipokines such as leptin, the levels of adiponectin in the plasma correlate inversely with adiposity and directly with insulin sensitivity [112, 113, 117, 118]. As such, high adiponectin concentrations in the plasma are needed to perform normal physiological actions in the cardiovascular system.

### 3.1. Adiponectin Signaling

Adiponectin possesses an oligomeric form [119, 120] which correlates with its physiological activities and consequently attracts further characterization and elaboration of its structure [119]. The gene that encodes for human adiponectin is present on chromosome 3q27 [121], a locus that is associated with diabetes and other CVDs [120, 122].

Adiponectin can form a wide range of multimer complexes and exists in three oligomeric forms: a low molecular weight (LMW) trimer, a middle molecular weight (MMW) hexamer, and a high molecular weight (HMW) multimer of 12–18 monomers [123–125]. The HMW complex is the functional unit and induces anti-inflammatory, antiatherogenic, and antidiabetic effects that protect against cardiovascular and metabolic disorders [120]. Adiponectin binds to a number of receptors, most importantly the adiponectin receptors (AdipoR1 and AdipoR2) and T-cadherin.

Different mice models with targeted deletion of* AdipoR1* or* AdipoR2* genes are defective in exhibiting adiponectin actions, indicating the important role of AdipoR1 and AdipoR2 in mediating adiponectin signaling. AdipoR1 is ubiquitously expressed, including the cardiovascular system, whereas AdipoR2 expression is high in the liver [126]. AdipoR1 is expressed highly in the heart compared to AdipoR2 [126]. Upon binding to its receptors, adiponectin activates several signaling pathways, such as AMPK and PPAR-*α*, and modulates gluconeogenesis and fatty acid oxidation [127, 128]. Indeed, several preclinical studies demonstrated the important role of AdipoRs in enabling adiponectin to carry out its physiological and metabolic functions [129–131]. Deletion of AdipoR1 blocked the adiponectin-mediated phosphorylation of AMPK, while AdipoR2 gene deletion increased adiposity and glucose intolerance, presumably due to increased gluconeogenesis [129]. AdipoR2-null mice demonstrated a hindered adiponectin-mediated PPAR-*α* activation and unlike AdipoR1-deficient mice showed resistance to diet-induced glucose intolerance [130]. However, deleting both receptors led to insulin resistance and glucose intolerance [129].

T-cadherin has also been recognized as an adiponectin receptor [132]. T-cadherin is present in adiponectin-targeted sites including the heart, VSMC, and endothelial cells [133]. It has been hypothesized that both AdipoRs and T-cadherin can act together to regulate adiponectin signaling in certain cells and tissues [120]. T-cadherin has been found as a crucial factor for adiponectin mediated cardioprotection in preclinical mice studies. Indeed, it was found that the expression of T-cadherin was abundant in the myocardium where it provided protection from pathological cardiac remodeling induced by stress [134]. T-cadherin-null mice showed an increased adiponectin level in the blood due to the diminished binding of adiponectin to its receptor sites on the heart.

### 3.2. Adiponectin and the Cardiovascular System

**Figure 2:**
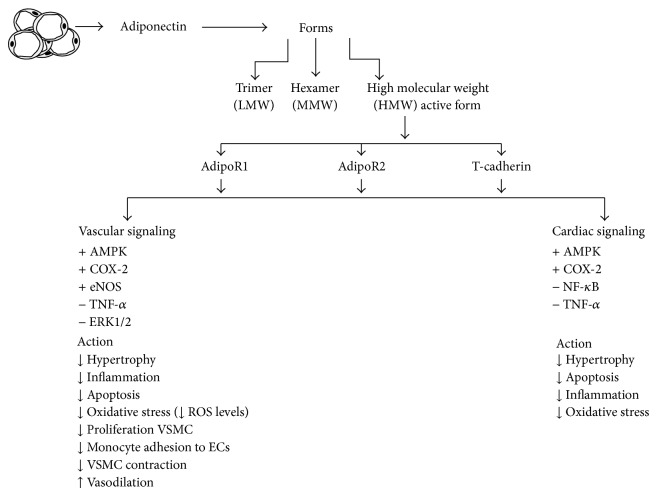
Summary of molecular signaling responses to adiponectin and their cardiac and vascular effect. ↑ or + represents activation of protein or effect whereas ↓ or − indicates inhibition. See text for more information.

Data obtained from different studies using both animal models and* in vitro* studies have demonstrated the multiple beneficial effects of adiponectin on the cardiovascular system, through direct and indirect actions on both cardiac and vascular cells (Figure 2).

#### 3.2.1. Cardiac Actions of Adiponectin

Left ventricular hypertrophy and its progression result from structural and functional cardiac disorders impairing the heart's ability to fill up with blood easily or to pump blood out efficiently; in both cases the body does not receive enough blood to meet its demands, hence interfering with its function. Several signaling pathways influence the cardiac hypertrophy manifestation including ROS formation, MAPK, and AMPK pathway activation [135, 136]. Adiponectin plays an important role in protection against cardiac remodeling by attenuating myocardial hypertrophy [137]. However, extensive knowledge about the mechanisms involving the relationship between low adiponectin levels and the development and progression of cardiac hypertrophy is still lacking and requires further examination.

Myocardial infarction (MI) is one of the primary causes of heart failure [138]. The heart usually responds to MI through “cardiac remodeling” by changing the shape, size, and function of the heart at the infarct site [139]. Treatment with exogenous adiponectin significantly reduced the MI size in mice hearts subjected to ischaemia/reperfusion. T-cadherin was shown to mediate the protective role of adiponectin against ischaemia/reperfusion cardiac injury [134]. This protective action was linked to the attenuation of ROS levels and TNF-*α* and the activation of AMPK and COX-2 [140]. Studies revealed that, under physiological condition, adiponectin exerts its beneficial effects via increased NO production from eNOS. However, under pathological states, adiponectin inhibits iNOS and thus decreases NO release and promotes cardiac injury [141].

#### 3.2.2. Vascular Actions of Adiponectin

In addition to its effects on cardiomyocytes, many studies demonstrated that adiponectin acts directly on vascular system and has protective effects against different vascular disorders, such as endothelial dysfunction [142], atherosclerosis [143], and hypertension [144].

Adiponectin initiates AMPK-mediated eNOS activation leading to NO production [116]. This action showed important physiological implications in the vasculature hemostasis [145–149]. Indeed, through NO, adiponectin was shown to exert many physiological actions on the vascular system, such as prevention of atherosclerosis, inhibition of VSMC proliferation, and regulation of vascular contraction and blood pressure [143].

Moreover, adiponectin selectively binds to different growth factors, such as heparin-binding epidermal growth factor-like growth factor and platelet-derived growth factor BB, thus attenuating their binding to their receptors [150, 151]. AdipoRs are expressed in platelets, and* in vitro* studies performed on human platelets showed that adiponectin inhibits platelet aggregation following collagen induction [143].

Another important function of adiponectin is its anti-inflammatory effect which is attributed to its ability to activate AMPK and other non-AMPK mechanisms. This leads to the inhibition of NFk*β* and consequently reduces the expression of adhesion molecules and the release of IL-8 following TNF-*α* stimulation [116, 152, 153].

## 4. Summary

Leptin is associated with obesity and is a potential contributor to many of the cardiovascular risks linked to obesity. It promotes hypertension, vascular remodeling, ROS generation, angiogenesis, atherosclerosis, and sympathetic nervous system stimulation (see Figure 1). Studies have shown that this hormone can predict myocardial infarction independently of conventional risk factors [154], and it is an independent predictor of myocardial infarction in patients with arterial hypertension [155]. On the other hand, leptin has been shown to have protective actions on the cardiovascular, renal, and gastric systems in ischemia/reperfusion injury, so labeling leptin as a strictly harmful hormone is not quite fair. Obese individuals are resistant to leptin, so some might argue that the leptin-associated harm on the cardiovascular system is due to leptin's inability to elicit its effects appropriately. Hence, further studies need to be done on leptin and specifically leptin resistance, in order to better understand leptin's function in obesity and promotion of cardiovascular diseases.

Leptin's role in developing LVH has been under scrutiny and controversy. While some researchers studied and demonstrated that leptin could possibly attenuate and reverse LVH, most studies have shown that leptin actually contributes to LVH progression. Several factors of LVH have been attributed to leptin, such as activation of the PI3K-AKT pathway and the MAP kinases ERK 1/2 and p38. Leptin's action on the sympathetic nervous system could also contribute to the development of LVH. Further studies need to be done on leptin's role in LVH in order to fully explain the pathophysiology of this form of myocardial remodeling. It is enticing to study whether leptin could play a therapeutic role in the myocardium in cases of heart failure and ischemia.

Studies have revealed that adiponectin preserves the normal physiology of the heart by protecting the heart and blood vessels against atherosclerosis, inflammatory, and oxidative stress. With further studies focusing on adiponectin's beneficial actions, this protein holds potential as new pharmacological therapy in cardiovascular disease.
